# Mobile Phone-Based Unobtrusive Ecological Momentary Assessment of Day-to-Day Mood: An Explorative Study

**DOI:** 10.2196/jmir.5505

**Published:** 2016-03-29

**Authors:** Joost Asselbergs, Jeroen Ruwaard, Michal Ejdys, Niels Schrader, Marit Sijbrandij, Heleen Riper

**Affiliations:** ^1^ Faculty of Behavioural and Movement Sciences Section Clinical Psychology Vrije Universiteit Amsterdam Amsterdam Netherlands; ^2^ EMGO Institute for Health Care and Research VU University Medical Centre Amsterdam Netherlands; ^3^ Mind Design Amsterdam Netherlands; ^4^ GGZ inGeest Amsterdam Netherlands; ^5^ Health and Life Sciences Faculty Telepsychiatry Unit Southern Denmark University Odense Denmark

**Keywords:** affect, data mining, ecological momentary assessment, experience sampling, mobile phone sensing

## Abstract

**Background:**

Ecological momentary assessment (EMA) is a useful method to tap the dynamics of psychological and behavioral phenomena in real-world contexts. However, the response burden of (self-report) EMA limits its clinical utility.

**Objective:**

The aim was to explore mobile phone-based unobtrusive EMA, in which mobile phone usage logs are considered as proxy measures of clinically relevant user states and contexts.

**Methods:**

This was an uncontrolled explorative pilot study. Our study consisted of 6 weeks of EMA/unobtrusive EMA data collection in a Dutch student population (N=33), followed by a regression modeling analysis. Participants self-monitored their mood on their mobile phone (EMA) with a one-dimensional mood measure (1 to 10) and a two-dimensional circumplex measure (arousal/valence, –2 to 2). Meanwhile, with participants’ consent, a mobile phone app unobtrusively collected (meta) data from six smartphone sensor logs (unobtrusive EMA: calls/short message service (SMS) text messages, screen time, application usage, accelerometer, and phone camera events). Through forward stepwise regression (FSR), we built personalized regression models from the unobtrusive EMA variables to predict day-to-day variation in EMA mood ratings. The predictive performance of these models (ie, cross-validated mean squared error and percentage of correct predictions) was compared to naive benchmark regression models (the mean model and a lag-2 history model).

**Results:**

A total of 27 participants (81%) provided a mean 35.5 days (SD 3.8) of valid EMA/unobtrusive EMA data. The FSR models accurately predicted 55% to 76% of EMA mood scores. However, the predictive performance of these models was significantly inferior to that of naive benchmark models.

**Conclusions:**

Mobile phone-based unobtrusive EMA is a technically feasible and potentially powerful EMA variant. The method is young and positive findings may not replicate. At present, we do not recommend the application of FSR-based mood prediction in real-world clinical settings. Further psychometric studies and more advanced data mining techniques are needed to unlock unobtrusive EMA’s true potential.

## Introduction

In mental health studies, researchers commonly rely on self-report questionnaires to follow the course of patients’ clinical symptoms [1]. However, these instruments are limited. They are retrospective and, therefore, susceptible to recall bias [2]. In addition, they are typically administered in clinical settings, which limit the degree to which measurements can be generalized to everyday life [3]. To address these limitations, there has been growing interest in so-called ecological momentary assessment (EMA), in which psychological phenomena are repeatedly assessed within patients’ natural environments [4,5].

Ecological momentary assessment includes various data collection methods and strategies, such as diaries and paper-and-pencil questionnaires. More recently, EMA consists of questions appearing on one’s mobile phone that need to be completed/answered at prompted time points, often multiple times a day (eg, ”on a scale from 1 to 10, how would you rate your level of irritation in the past 30 minutes?”) or immediately after a specific event of interest has occurred (eg, making a record in one’s diary or pressing a button on one’s mobile phone when experiencing a negative thought) [1]. As such, EMA reduces recall bias and increases the ecological validity of measurements, allowing researchers to better capture the dynamics of behavioral and emotional processes in everyday life (eg, [6,7]).

Despite EMA’s obvious advantages over retrospective questionnaires, self-report EMA—the dominant form in EMA research—still depends on explicit respondent input. This does not remove systematic biases such as social desirability. Furthermore, this dependence on explicit respondent input limits the amount of information that can be captured because study participants are usually willing to answer only a limited number of questions per day. When EMA is applied for too long, the cumulative response burden may negatively affect the validity of the measurements (eg, through reactivity) and response rates; for example, EMA compliance rates have been shown to erode significantly after 2 weeks of data collection [8]. These aspects limit the applicability of EMA in clinical practice and research.

Explicit respondent input may not be a necessary requirement of EMA because traces of behavior and experiences are already reflected in the log files of the technological devices that we use in everyday life. In what might be called “unobtrusive” EMA, hardware and software sensors embedded within mobile phones are used as unobtrusive monitors of user behavior (eg, physical activity, social activity) and contexts such as work or at home. Unobtrusive EMA silently samples data on a patient’s mobile phone. Data are collected, continuously if useful, without the need to constantly prompt the patient, thus minimizing the response burden and biases related to explicit respondent input. As such, unobtrusive EMA holds promise as an EMA variant, enabling rich data to be collected over longer periods of time. Of course, unobtrusive EMA cannot directly tap mental states. However, it may be useful for monitoring proxies of mental health (ie, variables that are theoretically associated with mental health), such as physiological states, behavioral patterns (ie, activity, social interactions), and contextual triggers (ie, specific locations or social environments). For instance, mobile phone context sensing has been explored in relation to alcohol dependence [9], academic performance [10], and depression [11,12].

In a pioneering study, LiKamWa et al [13] explored personalized regression modeling to predict day-to-day fluctuations of self-monitored mood from unobtrusively collected proxy variables of social activity, physical activity, and general mobile phone use. With mobile phone-logged data collected from 32 participants over 2 months, they found the predictive accuracy of personalized models to be high. Up to 93% of self-reported mood scores were correctly predicted within a tolerated error margin. These results suggest that mood studies could potentially follow participants longer by reducing the assessment burden of study participants through a mix of self-report EMA and unobtrusive EMA. Intrigued by this, we conducted a pilot replication study to further explore the feasibility of unobtrusive EMA-based mood prediction and to gain a better understanding of the challenges associated with collecting and processing unobtrusive EMA data and personalized predictive regression modeling.

## Methods

### Design and Study Procedures

**Figure 1:**
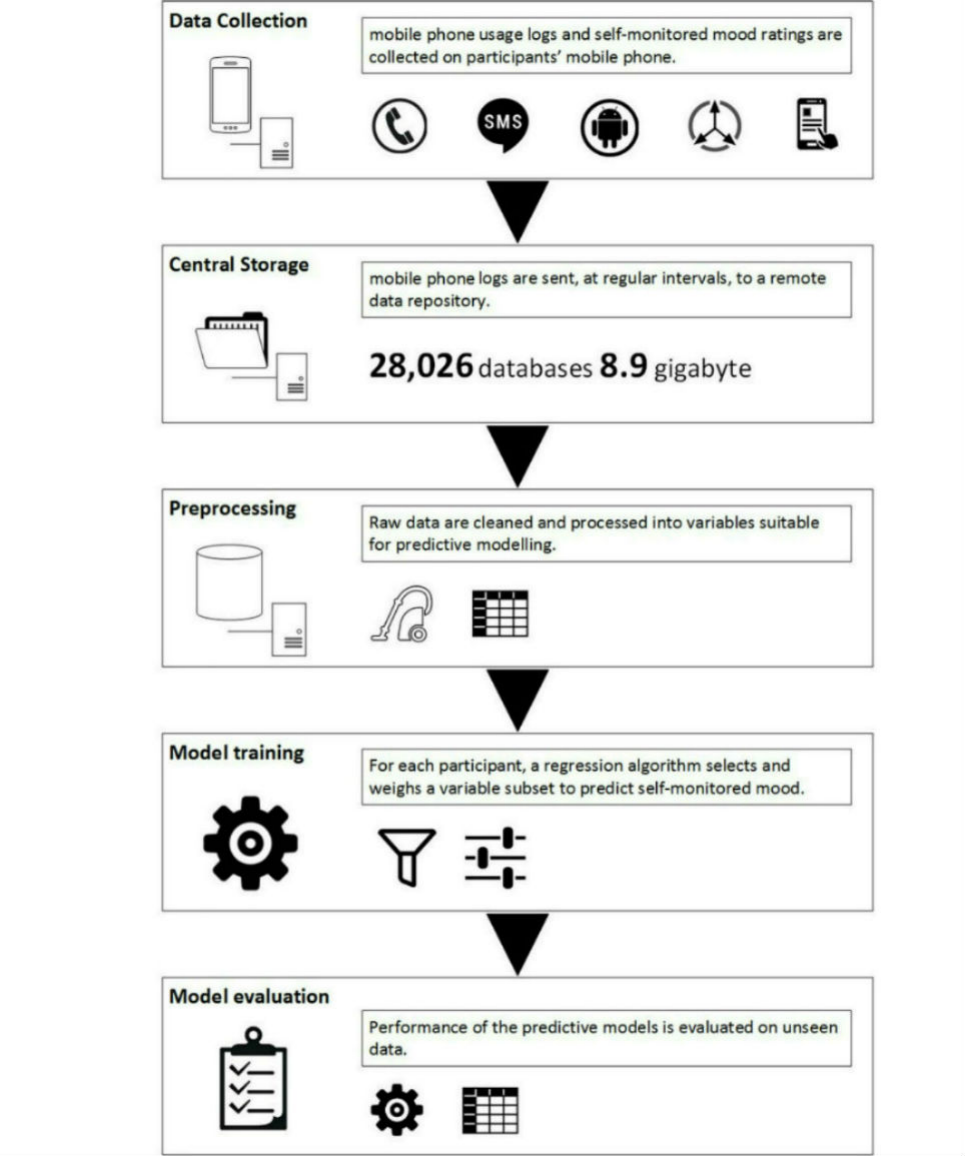
Overview of study setup.

This was an explorative uncontrolled pilot study, replicating the methods of LiKamWa et al [13]. A small group of Dutch university students (N=27) self-monitored their mood on their mobile phones for 6 weeks. Meanwhile, a faceless mobile phone app unobtrusively collected proxy variables of social activity, physical activity, and general mobile phone use from mobile phone sensors and app logs. Data collection was followed by a predictive modeling study, in which we checked whether personalized regression models could accurately predict day-to-day fluctuations of self-monitored mood from the unobtrusively collected mobile phone variables (see Figure 1). Primary study data and the R-script used for analysis are available for download (see Multimedia Appendices 1 and documentation and the R-studio project ZIP file in Multimedia Appendix 3).

### Participants

Through information flyers, we invited students from the campus of the Vrije Universiteit Amsterdam, the Netherlands, to take part in this study. Inclusion criteria for participation were (1) age 18 years or older and (2) owning an Android smartphone (minimal OS 2.2) that would be in use as the primary mobile phone during the study period (6 weeks). Applicants were screened for depressive symptoms before the study because we did not want to include participants with severe levels of depression. For this, we used the Center for Epidemiologic Studies Depression Scale (CES-D), which is a validated and often used 20-item questionnaire to assess past-week depressive symptoms [14] (Dutch Version [15]). CES-D total scores range between 0 and 60, with scores greater than 16 signaling mild depressive symptoms and scores greater than 27 signaling clinical depressive symptoms [16,17]. Applicants reporting clinical depression symptoms (ie, CES-D>27) were excluded from the study and referred to their general practitioner. To encourage students to take part in our study, we offered them a monetary reward depending on EMA response rates (ie, EMA response rates ≥50%: €20; rates ≥75%: €35; rates ≥95%: €47.50). Approval for the study was obtained from both the Science Committee and the Research Ethics Committee of the Psychology Department of the Vrije Universiteit Amsterdam (reference number: VCW.1311.016). All participants signed an informed consent form.

### Measures and Materials

#### Ecological Momentary Assessment of Mood

To collect self-monitored mood data (the target of the prediction task), we used eMate, an EMA mobile phone app developed at the Vrije Universiteit Amsterdam. This app prompted participants to rate their mood on their smartphone at five set time points per day (ie, approximately 09:00, 12:00, 15:00, 18:00, and 21:00). As in the study by LiKamWa et al [13], we assessed mood through the circumplex model of affect [18], which conceptualizes mood as a two-dimensional construct comprising different levels of valence (positive/negative affect) and arousal. Levels on both dimensions were tapped on a 5-point scale scored from -2 to 2 (low to high). Because recent studies suggest that single-item mood measures can provide predictive information on the development of depressive symptoms (eg, [19]), we also added a one-dimensional mood question, which asked participants to rate their current mood on a 10-point scale, with 1 as the negative and 10 as the positive pole.

#### Unobtrusive Ecological Momentary Assessment of Mood Predictors

For unobtrusive assessment, we developed iYouVU, a faceless mobile phone app based on the Funf open-sensing-framework [20,21] and prior research into communication habits based on mobile phone data collected without the user’s full awareness [22]. This app runs in the background, unnoticeable to the user, to collect designated sensor data and app logs. The app logged call events (ie, time/date of the call, duration, and contact of both incoming and outgoing calls), short message service (SMS) text message events (ie, time/date and contact), screen on/off events (ie, time/date), app use (ie, what app was launched, when, and for how long), and mobile phone camera use (ie, the time/date a picture was taken). All collected sensitive personal data, such as contact details (names, phone numbers), were anonymized during data collection by the app through the built-in cryptographic hash functions of the Funf framework. At set intervals during each day, and only when participants’ mobile phones were connected to Wi-Fi, the app sent collected data over the Internet to a remote central data server, in chunks of approximately 5 to 10 MB per data file.

We could not—or, in some cases, decided not to—monitor some of the features collected by LiKamWa et al [13]. Funf did not provide access to the metadata and content of email messages, so we could not include these variables in our study. Because Funf hashed browser history entries, we were not able to cluster the website domain. Furthermore, in a preliminary test, browser URL history did not function well enough to provide useful data; for that reason, we could not include this variable.

This was the first study with our experimental setup; therefore, we decided against collecting global positioning system (GPS) location data because we wanted to confirm adequate functioning of the technical setup before collecting highly sensitive personal data. Instead, as a proxy of activity, we collected accelerometer data.

### Data Preprocessing and Feature Engineering

Raw EMA and unobtrusive EMA data were preprocessed into a data file that summarized each day of each participant in a row of 53 variables, as described subsequently.

### Prediction Targets: Ecological Momentary Assessment Mood

As in the LiKamWa et al study [13], EMA data (ie, both the one-dimensional mood measure and the two measures of the circumplex model, valence and arousal) were aggregated to daily averages as targets for the mood prediction algorithms. Daily averages were standardized within each participant (ie, using means and standard deviations calculated for each participant separately).

### Mood Prediction Feature Set

Raw unobtrusive EMA data were aggregated into daily summaries and from these daily summaries we derived the following features as presented in Table 1.

**Table 1:** Mood prediction feature set.

Raw data and feature		Variables, n	Range
**Calls**			
	Caller top 5 contact frequency, 3-day window, normalized	5	0-1
	Caller top 5 contact duration, 3-day window, normalized	5	0-1
**SMS text message**			
	SMS text message top 5 contact frequency, 3-day window, normalized	5	0-1
**Accelerometer**			
	Percentage of high activity	1	0-1
**Screen**			
	Frequency of screen-on events (normalized within participant data)	1	–3 to 3^a^
	Total screen duration events (normalized within participant data)	1	–3 to 3^a^
**Apps**			
	Top 5 apps usage frequency, normalized	5	0-1
	Top 5 apps usage duration, normalized	5	0-1
	Categorized apps, usage frequency, normalized	11	0-1
	Categorized apps, usage duration, normalized	11	0-1
**Images**			
	Number of images taken (normalized within participant data)	1	0-1
**Mood history**			
	Mood of yesterday, standardized	1	–3 to 3^a^
	Mood of day before yesterday, standardized	1	–3 to 3^a^

^a^ Standard normal distribution (ie, 99.7% of values ranging between –3 and 3).

For phone calls and SMS text messages, we counted the number of interactions participants had with their five most frequent contacts. Following LiKamWa et al [13], we created a histogram of this interaction frequency over a 3-day history window and used the normalized frequency count as samples in the feature table. Similarly, we created a normalized 3-day histogram of call durations with the top five contacts. Most participants interacted only incidentally with persons outside their top five through calls or SMS text messages. Therefore, we limited the histograms to the five most frequently interacted contacts, in contrast to LiKamWa [13], who monitored the top 10 contacts. Altogether, raw call/SMS text message data were summarized into three predictive features (top five call frequency and duration and top five contact SMS text message frequency), comprising 15 variables.

Raw mobile phone screen on/off events were transformed into two features: (1) the total number of times the screen was turned on per day and (2) the total amount of screen time per day (calculated as the differences between the times of the screen on/off events). Both features were transformed to standard normal variables within each participant.

Accelerometer data represents the acceleration of the smartphone on the x, y, and z planes. Acceleration was sampled for 5 seconds each minute (at sample frequencies estimated to vary from 20-200 Hz, as determined by the hardware and software characteristics of participants’ mobile phones). Raw data were summarized (on the phone through Funf’s ActivityProbe) into a high activity variable by calculating the percentage of time at which the summed variance of the device’s acceleration (on the x, y, z planes) was above a set “high activity” threshold (ie, in which the summed variance exceeded 10 m/s^2^). These percentages were aggregated to the day level to provide an approximate measure of daily activity.

As daily measures of mobile phone app use, we created two 3-day normalized histograms for the daily frequency and duration of the five most frequently used mobile phone apps. In addition, we created normalized histograms of frequency and duration of the use of app categories. In accordance with the LiKamWa et al study [13], we categorized apps as either built-in, communication, entertainment, finance, games, office, social, travel, utilities, other, or unknown (11 categories). Categories of logged apps were determined through a scripted query of the Google Play Store. Apps that were unknown to the Google Play Store were manually categorized on the basis of an Internet search. In sum, the final dataset consisted of four features based on app usage logs: top five app frequency, top five app duration, app category frequency (11 categories), and app category duration (11 categories). These features resulted in 32 variables (5+5+11+11).

Phone camera logs were summarized to the number of photos taken per day. Next, this summary was transformed to the 0-1 scale for each participant separately by dividing all values by the maximum number of photos taken.

Finally, similarly to LiKamWa et al [13], we extended the predictive feature set with a simple representation of mood history, by adding lag 1 and lag 2 transformations of each mood variable (standardized within each participant).

In total, we derived a 53-dimensional variable set from 13 distinctive predictive features (Table 1). Because regression models are sensitive to large differences in the scales of independent variables, we transformed the scales of the variables to the standard normal distribution (ie, 99.7% of values ranging between –3 and 3). Interrelated variables (eg*,* top 5 call and top 5 app use) were normalized to the 0-1 range, following the methods of LiKamWa et al [13].

### Statistical Analysis

#### Personalized Predictive Model Training Algorithms

**Figure 2:**
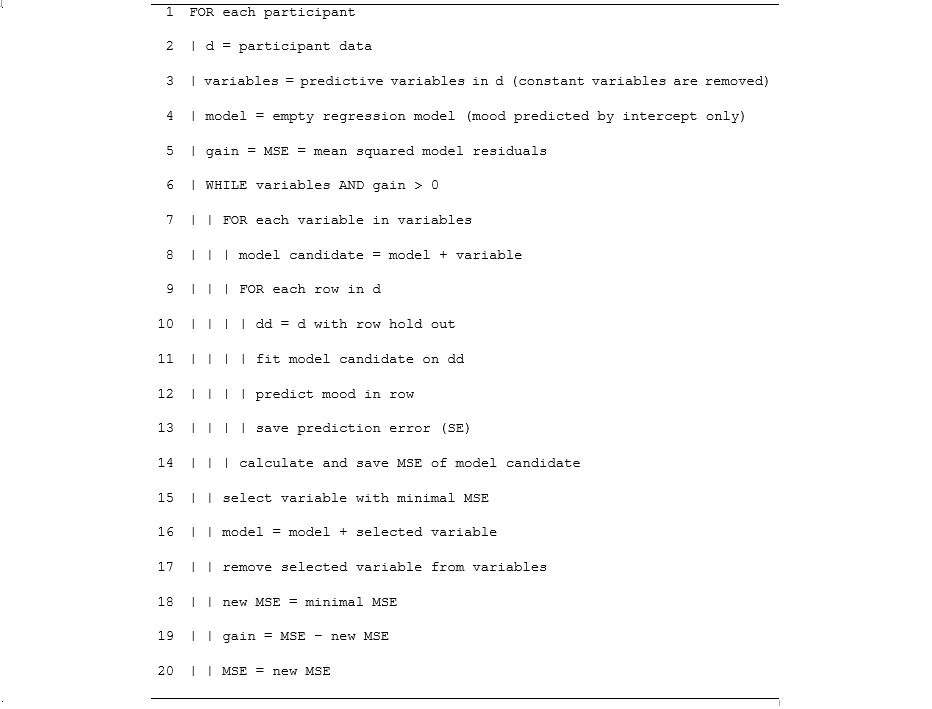
Predictive model building algorithm: forward stepwise regression with leave-one-out cross-validation.

Replicating LiKamWa et al [13], personalized mood prediction models were trained using forward stepwise regression (FSR), a multiple linear regression technique in which variables relevant to the prediction task are sequentially selected. We examined two FSR-variants: (1) the stepAIC procedure, as defined in the standard MASS toolbox of R [23], in which variables are selected on the basis of the Akaike information criterion (AIC) [24], and (2) the stepCV procedure, in which variables are selected based on their ability to minimize the cross-validated mean squared error. The algorithm of the second variant is outlined in Figure 2. For each participant, starting with the empty model (intercept only), the procedure sequentially adds, one by one, those predictive variables to the model that reduce the cross-validated mean squared error (MSE) the most until the MSE starts to increase. For the cross-validation in this algorithm, we used leave-one-out cross-validation (LOOCV), which was implemented by using the predicted residual sum of squares (PRESS) statistic on a single model run [25]. To prevent severe overfitting of regression models, we maximized the number of predictive variables in the models to the number of data points divided by 5 (ie, amounting to a maximum of eight variables with 42 data points).

### Predictive Performance Assessment

We assessed the predictive performance of the two FSR procedures through LOOCV. For each participant observation, we noted the differences between the observed mood rating and the mood rating predicted by the personalized FSR model trained on all other observations of that participant. Because we used both lagged mood prediction variables (ie, lag 1 and lag 2), we started to train our models from day 3 (ie, use a model trained on day 3:41 to predict day 42, etc). Thus, we assessed the degree to which the selected models could be generalized to unseen data.

We adopted two prediction performance measures. As a continuous measure, we used the cross-validated MSE (ie, the average squared difference between cross-validated predicted and observed scores). To compare results to those reported by LiKamWa et al [13], we also used a dichotomous “correct/incorrect” performance measure by recording whether cross-validated predicted scores fell within a preset tolerated error margin of 0.5 around observed scores.

### Benchmark Model Comparisons

To evaluate the personalized predictive regression models, we compared their performance to that of two naive benchmark prediction models that were agnostic of mobile phone usage data. The first benchmark model, the mean model, predicted the current mood state to be equal to the average observed mood state (ie, an intercept-only model). The second model, the history model, used a more dynamic time-series approach by assuming that the current mood state would be more similar to recent mood states. This was implemented as a linear regression model that included an intercept and two variables that represented the mood entries of the last 2 days (ie, the lag 1 and lag 2 of the dependent variable). Differences between the models in MSE and percentages of correct responses were tested for significance with the nonparametric Wilcoxon signed rank tests at a significance level of α=.05 because core assumptions of the parametric alternative (the paired *t* test) were not met.

### Incremental Predictive Performance Assessment

Using the full dataset (ie, 42 days) to predict mood at day 10 does not provide a valid test of how the algorithm would perform in an applied setting, in which increasing amounts of data become available over time. Therefore, we also assessed how trained models performed with increasing number of training days. For this, we applied the full training procedures iteratively on data from increasing numbers of training days (ie, day 4, 5, 41). For each number of training days, we tested how the trained models performed in predicting mood on the next day (ie, days 1 to 7 were used to predict mood on day 8, days 1 to 8 to predict mood on day 9, and so on). As we considered any attempt to predict scores on less than 7 days of data to be unfeasible, only mood scores between days 8 to 42 were included in this test. We hypothesized that the predictive performance of the models would increase with training sample size. We tested this by fitting a multiple regression model on the differences between the aggregated performance measures between the personalized models and the mean model (eg, with stepAIC MSE–mean model MSE as the dependent variable and the intercept and study day as independent variables). The intercept estimate of this model informed us on the comparative performance of the model at the start of the study period, whereas the study day regression parameter estimate informed us on the effect of increasing data points on the predictive performance of personalized models, in comparison to the most simple nonpersonalized benchmark model.

## Results

### Recruitment Process and Participants

In response to the recruitment information flyers, 42 students signed up for the study. Four participants scored above the CES-D cut-off (and were excluded) and five participants withdrew before collecting mobile phone data; therefore, 33 participants started the study. Mobile phone data of two participants did not reach the central study data server and data from four additional participants were not included in the analyses because these participants provided less than 20 days of complete data (ie, EMA and/or unobtrusive EMA data were missing on too many days to allow valid inferences). Thus, the final pruned dataset consisted of data from 27 participants. Table 2 shows the participants’ characteristics. Participants were young adults aged between 18 and 25 years. Mean CES-D screening score was 9.4 (SD 5.8, range 1-25). Three participants reported scores greater than 16, indicating mild depressive symptoms.

### Description of Ecological Momentary Assessment Measures

#### Ecological Momentary Assessment

In total, 4368 EMA mood ratings were collected. Of 27 participants, 18 (66%) provided mood ratings up to the study day 42 (range 28-42 days; mean 40.3, SD 3.3 days). Because some participants provided data intermittently, the mean number of days with valid data was 35.5 (SD 3.8). On sampled days, EMA schedule adherence, defined as the number of days on which participants contributed at least one mood rating to the dataset, was 88.80% (959/1080 days). On 91.9% (881/959) of sampled days, participants provided four or more ratings. EMA mood scores, on average, were neutral to positive (one-dimensional mood: mean 7.0, SD 0.95; valence: mean 0.7, SD 0.63; arousal: mean –0.1, SD 1.00) (Table 2).

#### Unobtrusive Ecological Momentary Assessment

Through the unobtrusive EMA mobile phone app, participants contributed 28,026 mobile phone log databases with a total disk size of 8.9 GB. Raw data logs detailed metadata of 5242 phone calls, 1800 text messages, 11,158 images, 22,973 hourly accelerometer-based activity summaries, 96,601 screen-on events, and 233,533 instances of app usage.

**Table 2:** Participant demographics, study adherence, and EMA summary statistics (N=27).

Measurements			Descriptive statistics
**Demographic characteristics**			
	Sex (female), n (%)		22 (78)
	Age (years), mean (SD)		21.1 (2.2)
	CES-D^a^ baseline score, mean (SD)		9.4 (5.8)
**EMA study adherence**			
	Number of days in study, mean (SD)		35.5 (3.8)
	Last day rated, mean (SD)		40.3 (3.3)
	Up to 42 days in study, n (%)		18 (67)
	**Responses per day (n), n (%)**		
		1	9 (1)
		2	25 (3)
		3	44 (5)
		4	228 (24)
		5	653 (68)
**EMA mood measures,** ^b^ **mean (SD)**			
	One-dimensional mood		7.0 (0.95)
	Circumplex: valence		0.7 (0.63)
	Circumplex: arousal		–0.1 (1.00)

^a^ CES-D: Center for Epidemiologic Studies Depression Scale (clinical cut-off: 16).

^b^ One-dimensional mood: 1-10 scale; circumplex-based mood: –2 to 2 scale.

### Predictive Performance of Personalized Models

#### Leave-One-Out Cross-Validation Results

**Figure 3:**
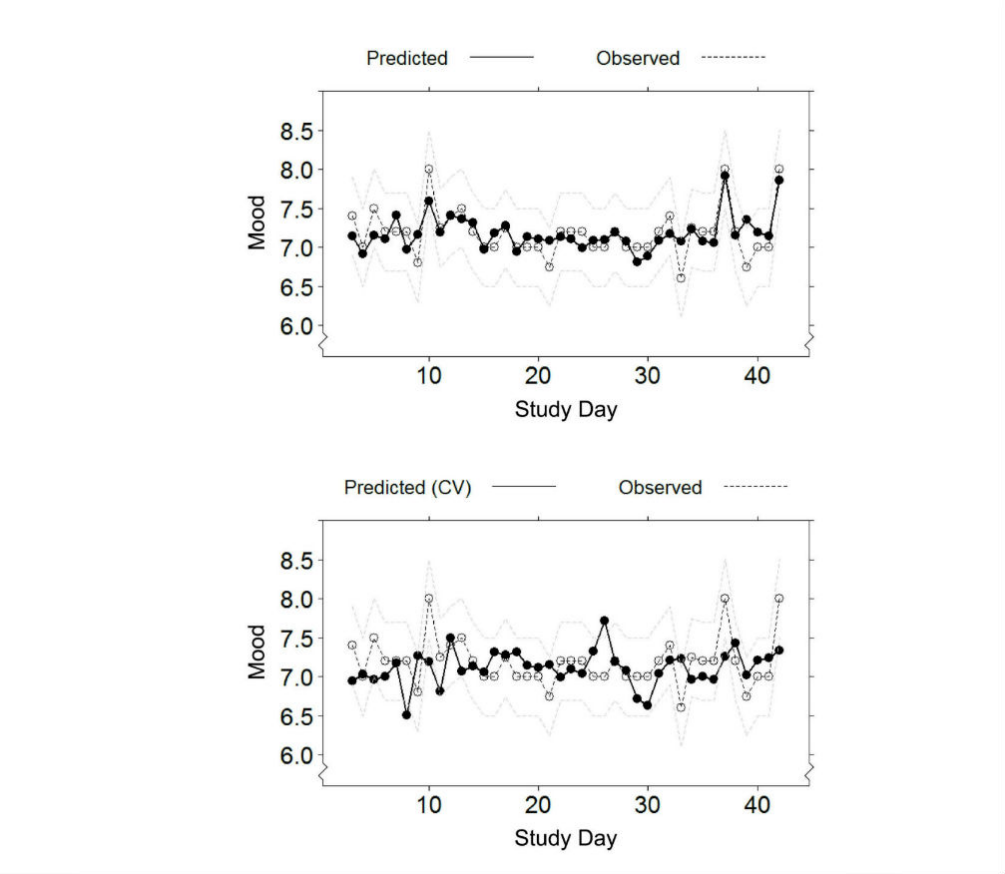
Observed versus predicted daily (one-dimensional) mood mean (range 1-10) for one participant with a personalized model trained on data including the predicted day (top) or excluding the predicted day (bottom) from the training procedure (ie, in-sample vs out-of-sample performance, respectively).

Figure 3 shows observed versus predicted responses of a representative participant, for days 3 to 42, without cross-validation (in-sample; top) and with cross-validation (out-of-sample; bottom). As expected, prediction errors were larger with cross-validation.

**Figure 4:**
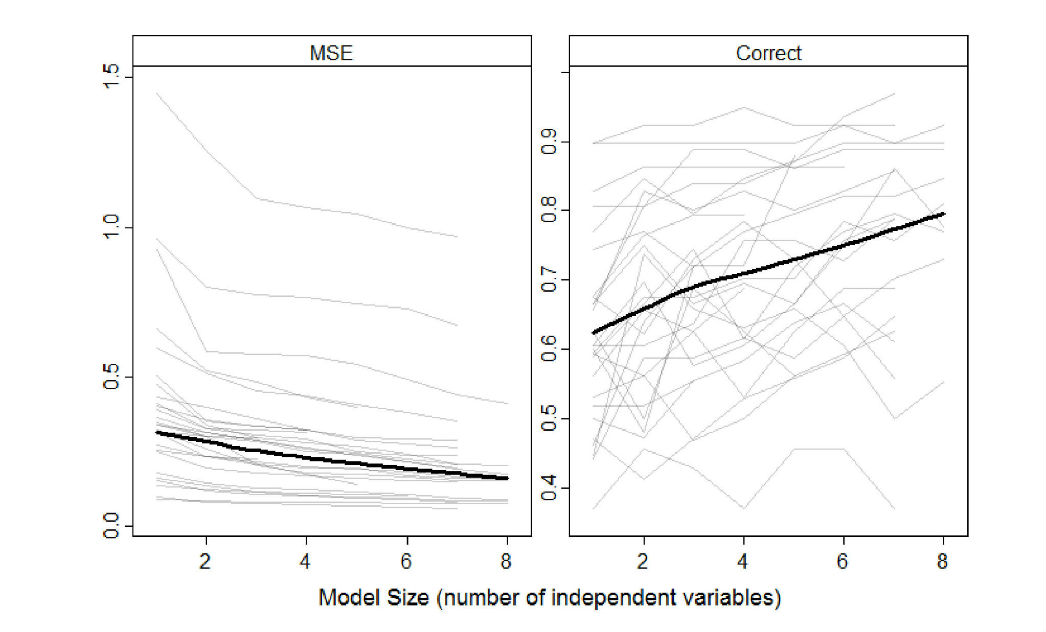
Predictive performance (mean squared error and % correct predictions) of the personalized models as observed for the prediction of the one-dimensional EMA mood measure for each participant (N=27) during cross-validated forward selection regression (stepCV).

Figure 4 shows the development of the cross-validated MSE and the percentage of correct responses during the stepCV training process, in which variables were sequentially added to the personalized model for each participant. With 42 days of training data, up to eight variables were selected (ie, the preset maximum of variables was reached). Governed by the algorithm, the MSE gradually decreased with each added variable. With regard to the correct predictions, the percentages tended to increase with increasing model complexity as well, but not continuously and not for each participant. This was expected because the percentage of correct predictions was not a parameter in the model optimization process.

Table 3 summarizes the predictive performance of the personalized models and the benchmark models, when trained on 42 days of data. Averaged over all participants, the percentage of correct cross-validated predictions of the personalized models ranged from 55% to 76%. Consistently, however, personalized model predictions were significantly inferior to those of naive benchmark models (all Wilcoxon signed rank test comparisons of differences in both performance measures were *P*<.02 in favor of the benchmark models). Compared with personalized models, the naive models improved the percentage of correct predictions by 5% to 9%. With regard to MSE, these improvements ranged from 0.07 to 0.27.

**Table 3:** Predictive performance of personalized models and naive benchmark models.^a^

Model^b^	One-dimensional mood, mean (95% CI)^c^		Multidimensional mood (circumplex), mean (95% CI)^c^			
	Correct	MSE	Valence		Arousal	
			Correct	MSE	Correct	MSE
Step CV	57% (50%-64%)	0.67 (0.35-0.98)	76% (71%-81%)	0.22 (0.17-0.27)	54% (47%-61%)	0.58 (0.40-0.76)
Step AIC	55% (49%-61%)	0.58 (0.41-0.75)	76% (71%-81%)	0.23 0.17-0.29)	55% (49%-61%)	0.58 (0.42-0.74)
Mean	62% (56%-68%)	0.41 (0.30-0.52)	85% (81%-89%)	0.15 0.12-0.18)	63% (57%-69%)	0.34 (0.27-0.41)
History	64% (58%-70%)	0.40 (0.29-0.51)	83% (79%-87%)	0.15 (0.12-0.18)	63% (58%-68%)	0.33 (0.27-0.39)

^a^ Results shown are those obtained with 42 days of training data for N=27 participants.

^b^ In personalized prediction models, Step CV and Step AIC*,* multiple regression models were constructed through stepwise forward variable selection based on cross-validated MSE (see Figure 2) and the Akaike information criterion (AIC), respectively. The mean model included the intercept only and the history model included the intercept and mood at T1 and T2.

^c^ The MSE column shows the mean of the (cross-validated) squared prediction residuals, and the correct column shows the percentage of predictions that fell within the tolerated error margin around the observed score (ie, cross-validated residual ≤0.5). All differences between the performance criteria of the personalized model approaches and the benchmark models were significant (Wilcoxon signed rank tests: *P*<.02).

### Incremental Performance

**Figure 5:**
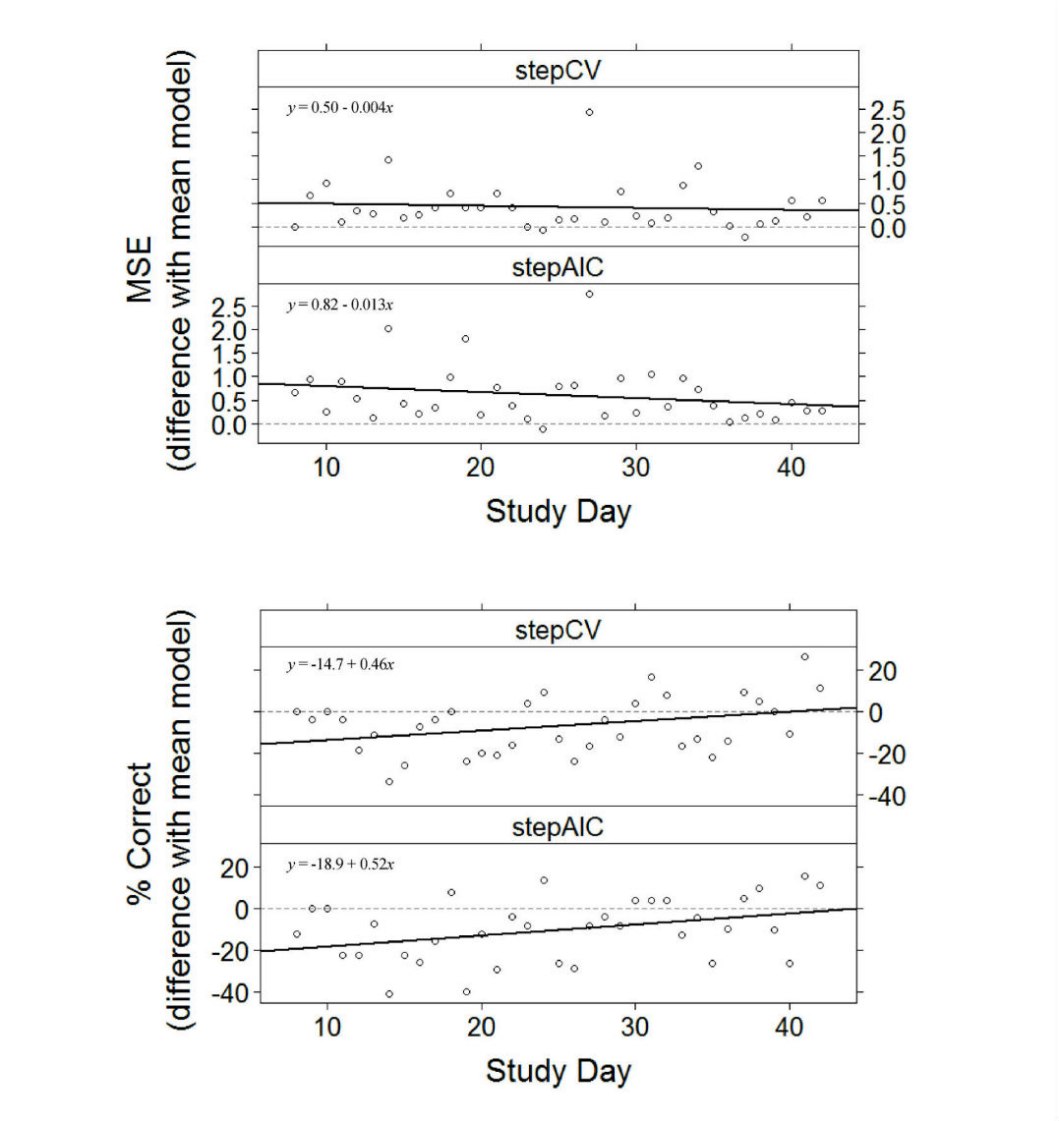
Relative predictive performance of stepAIC and stepCV (in comparison to the intercept-only model) as a function of increasing training data size (one-dimensional mood).

In the incremental test, predictive models were built using increasing days of training data. After 1 week, data up to day d–1 were used to predict mood from unseen data collected at day d (ie, days 1 to 7 to predict mood at day 8, days 1 to 8 at day 9). As shown in Table 4 (for all outcomes), and illustrated by Figure 5 (for one-dimensional mood), MSE was predominantly higher in the personalized models in comparison to the MSE of the mean model across the study period*,* whereas the percentage of correct responses was lower. The predictive performance in both procedures improved slightly with increasing amounts of training data (ie, see the “study day” regression parameter estimates in Table 4). However, these improvements were significant for only 3 of 12 tests of the effect of study day on predictive performance (see Table 4).

**Table 4:** Relative predictive performance of the personalized models compared to the intercept-only benchmark regression model.^a^

Measure		MSE, b (SE)		% Correct, b (SE)	
		Intercept^b^	Study day	Intercept^b^	Study day
**Mood**					
	stepAIC	0.82 (0.20)	–0.0126 (0.0100)	–18.9 (4.7)	0.52 (0.24)^c^
	stepCV	0.50 (0.17)	–0.0045 (0.0086)	–14.7 (4.2)	0.46 (0.21)^c^
**Circumplex: valence**					
	stepAIC	0.94 (0.40)	–0.0261 (0.0201)	–21.2 (3.1)	0.34 (0.16)^c^
	stepCV	0.25 (0.08)	–0.0041 (0.0042)	–12.1 (3.0)	0.03 (0.15)
**Circumplex: arousal**					
	stepAIC	0.49 (0.12)	–0.0003 (0.0061)	–14.8 (3.0)	0.29 (0.15)
	stepCV	0.47 (0.13)	0.0002 (0.0064)	–10.2 (3.6)	0.02 (0.18)

^a^ Results show the estimated parameters of the linear regression model (ie, mood ~1 + “study day”); MSE: mean squared error; b: regression estimate (unstandardized); SE: standard error of regression estimate.

^b^ All intercept estimates were significant at α=.05.

^c^ These study day estimates were significant.

## Discussion

In this pilot study following up on previous research [13], we examined whether personalized regression models could accurately predict day-to-day fluctuations of self-monitored mood from mobile phone usage logs. We found that personalized regression models, trained through FSR, correctly predicted 55% to 76% of self-reported EMA mood ratings. However, the predictive performance of the FSR models was clearly inferior to that of naive nonpersonalized predictive benchmark models. The performance of the personalized models tended to improve over time. Nonetheless, within our 42-day study period, these improvements were not large enough to compensate for the poor relative predictive performance of the FSR approach.

### Comparison to Previous Findings

Our study provides a sobering adjunct to the study of LiKamWa et al [13], in which personalized FSR models predicted self-reported mood ratings with 93% accuracy (and also considerably higher than benchmark models). Their results implied that mood inference models could be successfully trained through FSR on personal unobtrusive EMA data. In our replication, by contrast, we found no clear benefits of the personalized feature selection approach over naive benchmark prediction models. Based on our results and Ockham’s law of parsimony (that the simplest of competing models should be preferred), we would recommend against FSR modeling on unobtrusive EMA data to predict short-term mood variation. To predict short-term mood variation, at least in the first 42 days and with study groups similar to ours, our results suggest that it would suffice to simply predict the mean of past responses.

Forward stepwise regression may not be the most suitable basis for automated predictive modeling of day-to-day mood fluctuations. Among other problems, the procedure is known to be vulnerable to overfitting (ie, the selection of overly complex models that perform poorly on unseen data) [26,27]. When the number of potential predictive variables exceeds the number of training samples, as was the case in our study, FSR appears to be too sensitive to random fluctuations in the training data and instability of the regression parameters, resulting in poor generalization to new data. We tried to avoid this by building LOOCV into a variant of the FSR model selection procedure (through the PRESS statistic); unfortunately, this did not improve outcomes.

It should be noted that the 93% predictive accuracy rate in the LiKamWa et al study [13] was observed with 60 days of data, whereas we only collected data for 42 days. If more training data had been collected, the performance of the FSR models might have been better. As stated, we found some evidence that the performance of the models improved slightly over time. But how many individuals would be willing to self-report mood ratings for 60 days? Our participants were paid and their adherence to the 42-day EMA self-report schedule was satisfactory probably for that reason. In real-life settings, however, we expect the assessment burden of continued self-report EMA to result in large dropout rates, even before day 42. LiKamWa et al [13] recognized this problem. They proposed a “hybrid personalized/all user” modeling approach as a solution, in which data of other users are used to construct a base predictive model, which is then tuned to individual data. This approach might reduce the required amount of training days. Unfortunately, due to time restrictions, we could not follow up on this interesting suggestion.

The different outcomes of our study and that of LiKamWa et al [13] may also be explained by differences in study populations (ie, Dutch students versus a predominantly Chinese student population). The performance of predictive algorithms may be sensitive to the cultural background of study respondents. Previous studies have found qualitative differences in the presentation of psychopathological symptoms in Chinese and Western participants [28]. In addition, we note that we excluded individuals with severe depressive symptoms from our study sample, whereas LiKamWa et al [13] did not. This possibly reduced the variance in the mood data somewhat, making it more difficult for the feature selection algorithms to select effective predictors. Some support for this explanation can be found in the similar results that were obtained with both benchmark models (ie, the history model, which added recent mood entries to the mean model, did not substantially improve the predictive performance). However, because only four eligible participants were excluded on the basis of this exclusion criteria, we feel that this difference cannot fully explain the divergent results. A new replication study in a clinical population would be informative to explore whether the predictive approach works better when mood variations are more salient.

Our study also differed from that of LiKamWa et al [13] in terms of the type of unobtrusive variables that were assessed. By using the Funf framework, we were not able to access detailed email data (which was identified as one of the more discriminative features in the LiKamWa study). Likewise, we could not adequately capture anonymized browser history data. Finally, we also refrained from collecting GPS location data. It is possible that the availability of these omitted features would have resulted in stronger predictive models. However, we doubt whether the primary finding of our study, namely the inferior predictive performance of the unobtrusive EMA regression models, would have been different if email, browser history, and GPS location data had been available. Even if these features had been included in our feature set, we fear that FSR’s proneness to overfitting would still result in the selection of models that would explain too much of the variation in the training data and too little of the variation in unseen data.

Finally, we should consider the reliability of the self-report EMA mood measures. The circumplex model is a common EMA mood measure and recent studies have suggested that single-item mood measures can provide predictive information on the development of depressive symptoms (eg, [19]). However, there certainly remains much to be learned with regard to the psychometric properties of single-item EMA measures in different populations and contexts. Therefore, readers are reminded that we cannot rule out the possibility that our results were negatively affected by noisy measurement of the day-to-day mood variations.

### Next Steps

We wish to stress that the sobering results of our study—in our opinion—do not dismiss the unobtrusive EMA method. On the contrary, based on our experiences, we would argue that mobile phone-based unobtrusive EMA is a technically feasible and potentially powerful assessment method to collect a continuous stream of objective patient data with little to no respondent burden. Mobile phone-based unobtrusive EMA requires innovative technology to capture mobile phone sensor data and send the data to a remote central storage server. Its technical requirements are complex. Despite this complexity, however, data collection was quite successful in this study. Thus, although we do not recommend real-world application of FSR-based mood predictions in the field, we do recommend further exploration and refinement of unobtrusive EMA methods.

Although conducting this study was instructive, we feel that substantial progress can and should be made with regard to unobtrusive EMA feature engineering. In our opinion, one of the reasons for the poor performance of the predictive models should be sought in the rather tentative and distant relationship between the included unobtrusive predictors and mood. Transforming raw data into meaningful features can significantly improve predictive power [29]. This was recently shown by Saeb et al [12], who transformed raw GPS data into several variables relevant to depression. Unprocessed, GPS data are not predictive of depressive symptoms. However, transformed into features that represent, for example, home stay, number of locations visited (clusters), or circadian movement patterns, Saeb et al [12] revealed significant correlations with a validated depression measure. If we relate this to our dataset, we might be able to transform raw accelerometer data into a circadian rhythm index to construct a more relevant feature. Personalized modeling and data mining are exciting fields. In the short term, however, most progress will probably come from taking one step back to construct meaningful, theoretically relevant features that can be derived from (combinations of) raw unobtrusive EMA data.

The aim of this study was to replicate the study of LiKamWa et al [13] as much as possible; therefore, we did not deviate from using FSR. For future feature selection/personalized modeling studies with unobtrusive EMA data, however, more advanced statistical techniques might have to be considered, such as time-series analysis (eg, [30]), regression trees, support vector machines, or LASSO/ridge regression [31]. The success of these statistical techniques will probably also depend on the availability of adequate training samples. From this perspective, we recommend against the aggregation of unobtrusive EMA data in future studies. By aggregating the unobtrusive EMA data into daily summaries, as we did in this study to replicate LiKamWa et al [13], one of the more interesting aspects of unobtrusive EMA measures, their semicontinuous sample frequency, was lost. With it, information was lost on processes occurring during the day and statistical power was greatly reduced. In short, we expect better results with unaggregated unobtrusive EMA data.

### Conclusion

Mobile phone-based unobtrusive EMA is a technically feasible and potentially powerful EMA variant that may be key to future advances in the study and treatment of psychiatric symptoms. However, the unobtrusive EMA method is young and positive experiences with early apps may not replicate. Forward stepwise regression appears to be too vulnerable to overfitting to accurately predict day-to-day mood fluctuations from aggregated unobtrusive EMA data. Based on our results, and in contrast to previous reports, we do not recommend the application of this modeling strategy in real-world clinical settings. To come up with more robust solutions, future studies should address feature engineering and explore alternative advanced data mining techniques.

## Appendix

Multimedia Appendix 1 Study data description.

Multimedia Appendix 2 R source code.

Multimedia Appendix 3 Zipfile R project containing the data and analyses used in this manuscript.

## Abbreviations

AIC: Akaike information criterion
CES-D: Center for Epidemiologic Studies Depression Scale
EMA: ecological momentary assessment
FSR: forward stepwise regression
GPS: global positioning system
LOOCV: leave-one-out cross-validation
MSE: mean squared error
PRESS: predicted residual sum of squares
SMS: short message service
