# Supplementary material for: Mobile Phone-Based Unobtrusive Ecological Momentary Assessment of Day-to-Day Mood: An Explorative Study
Source: J Med Internet Res. 2016 Mar 29;18(3):e72. doi: 10.2196/jmir.5505 (PMC4829730; doi:10.2196/jmir.5505)
Supplement: Multimedia Appendix 1 [file jmir_v18i3e72_app1.pdf]

# APPENDIX 1: Study Data Description

Primary Data file description of:

Smartphone-based Unobtrusive Ecological Momentary Assessment of Day-to-Day Mood: # An Explorative Study.

Joost Asselbergs, Jeroen Ruwaard, Michal Ejdys, Niels Schrader, Marit Sijbrandij & Heleen Riper

Corresponding author: j.a.g.asselbergs@vu.nl

| Variable Name | Variable Description |
|---|---|
| | **Participant identification** |
| id | subject id, factor with 27 levels (AS14.01 .. AS14.33) |
| | **Time** |
| time | day of year (in 2014, 1) |
| stime | days in study (3-42) |
| | **EMA Self-reports** |
| mood | EMA mood: uni-dimensional (1-10); daily average. |
| valence | EMA mood: circumplex two-dimensional, valence (-2 to 2); daily average. |
| arousal | EMA mood: circumplex two-dimensional, arousal (-2 to 2); daily average. |
| | **UEMA predictors** |
| | **Photo camera log** |
| image.n | number of photo's taken on smartphone. |
| **SMS log** | |
| sms.c1c | number of SMS's sent to top SMS contact #1 (normalised histogram). |
| sms.c2c | number of SMS's sent to top SMS contact #2 (normalised histogram). |
| sms.c3c | number of SMS's sent to top SMS contact #3 (normalised histogram) |
| sms.c4c | number of SMS's sent to top SMS contact #4 (normalised histogram) |
| sms.c5c | number of SMS's sent to top SMS contact #5 (normalised histogram) |
| **Call log** | |
| call.c1c | number of calls made to top call contact #1 (normalised histogram) |
| call.c2c | number of calls made to top call contact #2 (normalised histogram) |
| call.c3c | number of calls made to top call contact #3 (normalised histogram) |
| call.c4c | number of calls made to top call contact #4 (normalised |

| | |
|---|---|
| | histogram |
| call.c5c | number of calls made to top call contact #5 (normalised histogram) |
| call.c1d | duration of calls made to top call contact #1 (normalised histogram) |
| call.c2d | duration of calls made to top call contact #2 (normalised histogram) |
| call.c3d | duration of calls made to top call contact #3 (normalised histogram) |
| call.c4d | duration of calls made to top call contact #3 (normalised histogram) |
| call.c5d | duration of calls made to top call contact #4 (normalised histogram) |
| **Application launcher logs (top 5 apps)** | |
| app.a1c | number of times top app #1 was launched (normalised histogram) |
| app.a2c | number of times top app #2 was launched (normalised histogram) |
| app.a3c | number of times top app #3 was launched (normalised histogram) |
| app.a4c | number of times top app #4 was launched (normalised histogram) |
| app.a5c | number of times top app #5 was launched (normalised histogram) |
| app.a1d | duration of use of top app #1 (normalised histogram) |
| app.a2d | duration of use of top app #2 (normalised histogram) |
| app.a3d | duration of use of top app #3 (normalised histogram) |
| app.a4d | duration of use of top app #4 (normalised histogram) |
| app.a5d | duration of use of top app #5 (normalised histogram) |
| **Application launcher logs (categorised apps)** | |
| appCat.builtin.n | app use frequency (3-day normalised histogram) |
| appCat.communication.n | app use frequency (3-day normalised histogram) |
| appCat.entertainment.n | app use frequency (3-day normalised histogram) |
| appCat.finance. | app use frequency (3-day normalised histogram) |
| appCat.game.n | app use frequency (3-day normalised histogram) |
| appCat.office.n | app use frequency (3-day normalised histogram) |
| appCat.social.n | app use frequency (3-day normalised histogram) |
| appCat.travel.n | app use frequency (3-day normalised histogram) |
| appCat.utilities.n | app use frequency (3-day normalised histogram) |
| appCat.weather.n | app use frequency (3-day normalised histogram) |
| appCat.other.n | app use frequency (3-day normalised histogram) |
| appCat.unknown.n | app use frequency (3-day normalised histogram) |
| appCat.builtin.sum | app use duration (normalised histogram) |

| | |
|---|---|
| appCat.communication.sum | app use duration (normalised histogram) |
| appCat.entertainment.sum | app use duration (normalised histogram) |
| appCat.finance.sum | app use duration (normalised histogram) |
| appCat.game.sum | app use duration (normalised histogram) |
| appCat.office.sum | app use duration (normalised histogram) |
| appCat.social.sum | app use duration (normalised histogram) |
| appCat.travel.sum | app use duration (normalised histogram) |
| appCat.utilities.sum | app use duration (normalised histogram) |
| appCat.weather.sum | app use duration (normalised histogram) |
| appCat.other.sum | app use duration (normalised histogram) |
| appCat.unknown.n | app use duration (normalised histogram) |
| **Screen log** | |
| screen.duration | average screen duration, per screen-on moment (standardized within participant) |
| screen.n | screen-on frequency (standardized within participant) |
| **Accelerometer** | |
| accelerometer.high | average percentage of high accelerometer data points |
| **Lagged EMA self-report** | |
| mood.l1 | unidimensional mood (EMA), lag 1 |
| mood.l2 | unidimensional mood (EMA), lag 2 |
| valence.l1 | two-dimensional mood - valence (EMA circumplex), lag 1 |
| valence.l2 | two-dimensional mood - valence (EMA circumplex), lag 2 |
| arousal.l1 | two-dimensional mood - arousal (EMA circumplex), lag 1 |
| arousal.l2 | two-dimensional mood - arousal (EMA circumplex), lag 2 |
