# Supplementary material for: Mobile Phone-Based Unobtrusive Ecological Momentary Assessment of Day-to-Day Mood: An Explorative Study
Source: J Med Internet Res. 2016 Mar 29;18(3):e72. doi: 10.2196/jmir.5505 (PMC4829730; doi:10.2196/jmir.5505)
Supplement: Multimedia Appendix 2 [file jmir_v18i3e72_app2.pdf]

APPENDIX 2: R Source Code

```
##########################################################################################
##########
# Raw data / Analysis Appendix of:
#
# Smartphone-based Unobtrusive Ecological Momentary Assessment of Day-to-Day Mood: An
Explorative Study.
# Joost Asselbergs, Jeroen Ruwaard, Michal Ejdys, Niels Schrader, Marit Sijbrandij
# & Heleen Riper
#
# Joost Asselbergs / Jeroen Ruwaard, dec 2015
# Corresponding author: j.a.g.asselbergs@vu.nl

##########################################################################################
##########
# 1. Clear environment
rm(list = ls())

##########################################################################################
##########
# 2. ENSURE LIBRARIES
# NOTE: installs missing packages if needed

ensurePackage = function(pname) {
  opt = options("warn" = -1) # suppress warnings
  if (!(require(pname, character.only=TRUE, quietly=TRUE))) {
    install.packages(pname) # package is not installed; download an install
  }
  options(opt) # reset warnings
}

ensurePackage("dplyr")        # for bind_rows
ensurePackage("caret")        # for nzv
ensurePackage("MASS")         # for stepAIC
ensurePackage("lattice")      # for graphs
ensurePackage("latticeExtra") # for layering of lattice plots

##########################################################################################
##########
# 3. Options
# set working directory and lattice theme options

# Working directory
wd = "./"

# Lattice plotting options
ltheme <- canonical.theme(color = FALSE)      # in-built B&W theme
ltheme$fontsize = list(text = 32)             # default fontsize (recommended: 16;
presentations: 32)
ltheme$strip.background$col <- "transparent"  # strip bg
ltheme$add.text$cex = .8                      # strip fontsize
ltheme$layout.heights$strip = 1.2             # strip height
lattice.options(default.theme = ltheme)       # set as default
lattice.options(panel.error = function(e) {}) # no errors in panes


##########################################################################################
##########
# 4. Load aggregated study data (UEMA_MOOD data frame)
# data is pre-processed: 1 row per day per participants (see manuscript)

load(paste0(wd, "UEMA_MOOD.RData"))


##########################################################################################
##########
```

```
# 5. Focus analysis on one of three prompted EMA outcomes: mood, arousal or valence
# uncomment one of the sections below to focus the analyses on a specific outcome
variable

#focus on mood: remove lagged arousal / valence as predictors
dd = UEMA_MOOD[!names(UEMA_MOOD) %in% c("arousal.l1","arousal.l2", "valence.l1",
"valence.l2")]
dd = dd[complete.cases(dd), ]
OUTCOME = "mood"

# focus on valence: remove lagged arousal / mood
# dd = UEMA_MOOD[!names(UEMA_MOOD) %in% c("mood.l1","mood.l2", "arousal.l1",
"arousal.l2")]
# dd = dd[complete.cases(dd), ]
# OUTCOME = "valence"

# # focus on arousal: remove lagged mood / valence as predictors
# dd = UEMA_MOOD[!names(UEMA_MOOD) %in% c("mood.l1","mood.l2", "valence.l1",
"valence.l2")]
# dd = dd[complete.cases(dd), ]
# OUTCOME = "arousal"


#####################################################################################
#########
# 6. Define result record (PUBLISHED_ANALYSES.RDA)
# (will contain key data of analyses on all outcome measures after analyses)

# Load previously saved data (if these exist)
# ignore error if it occurs (if data is not found, it will be created).

load(paste0(wd,"PUBLISHED_ANALYSES.RDA"))

if(!exists("PUBLISHED_ANALYSES")) PUBLISHED_ANALYSES = list()
PUBLISHED_ANALYSES[[OUTCOME]] = list(outcome = OUTCOME) # note: starts fresh results
record for outcome


#####################################################################################
#########
# 7. Define Predictive Modelling Algorithms
#
# - FSR_stepAIC
# - FSR_stepCV
#
# NOTE: benchmark models (Mean/History) are defined in section 8

#
# Forward step-wise regression, by AIC (standard R lib, MASS Package)
# see ?stepAIC

# PARAMS:
# ddd    : participant data frame
FSR_stepAIC = function(ddd){

  # set feature set
  outcome = unlist(ddd[OUTCOME])
  d = ddd[7:ncol(ddd)]

  # remove variables with zero variance and
  # variables for which the ratio of the occurence of the most occuring and second-
most
  # occuring variable is less than 15
  nzv <- nearZeroVar(d, saveMetrics = TRUE)
  d = d[names(d) %in% names(d)[!(nzv$nzv | (nzv$freqRatio > 15))]]
  d$outcome = outcome

  require(MASS)
```

```
  scope = paste("~ 1 +", paste(names(d[1:(length(d)-1)]), collapse="+"))

  # we need step to make sure not too many predictors are selected
  max_vars = ceiling(nrow(d)/ 5)

  # if empty model explains everything, no need to run AIC (AIC -Inf)
  if(any(residuals(lm(outcome ~ 1, d)) > 0)){
    r = stepAIC(lm(outcome ~ 1, d), data = d, scope = list(upper = scope),
      direction = "forward", trace = 0, k = 3, step = max_vars)
    r = list(fit = lm(formula(r), d))
  } else {
    r = list(fit = lm(outcome ~ 1, d))
  }

  return(r)
}


#
# Forward step-wise regression, by crossvalidated mean squared error (PRESS)
# see manuscript.

# PARAMS:
# ddd    : participant data frame
# plot   : if TRUE, plot MSE/%correct trace
FSR_stepCV = function(ddd, plot = FALSE) {

  # set feature set
  outcome = unlist(ddd[OUTCOME])
  d = ddd[7:ncol(ddd)]

  # remove variables with zero variance and
  # variables for which the ratio of the occurence of the most occuring and second-
most
  # occuring variable is less than 15
  nzv <- nearZeroVar(d, saveMetrics = TRUE)
  d = d[names(d) %in% names(d)[!(nzv$nzv | (nzv$freqRatio > 15))]]
  d$outcome = outcome

  features = names(d)[1:ncol(d) - 1] # last column in dataset is outcome

  # start with empty model
  step  = 1
  sf    = 1
  model = "outcome ~ 1"

  # save empty model results
  fit        = lm(as.formula(model), d)
  hii        = ls.diag(fit)$hat
  PRESS      = sum( (fit$residual/(1-hii))^2) / nrow(d) # note: it's the mean.
  correct    = sum( (fit$residual/(1-hii))^2 <= .25) / nrow(d)
  PRESS_GAIN = 100

  # model size not allowed to exceed 5 obs per var
  max_step = ceiling(nrow(d)/ 5)

  # initialize result matrix
  r  = expand.grid(
    id      = ddd$id[1],
    step    = 1:max_step,
    PRESS   = NA,
    correct = NA,
    model   = NA)
  r[r$step == step, "PRESS"]   = PRESS
  r[r$step == step, "correct"] = correct
  r[r$step == step, "feature"] = "intercept"
  r[r$step == step, "model"]   = model
```

```
  # while relevant features, sequentially add new features to the model
  while(length(features) > 0 & PRESS_GAIN > 0.00 & step < max_step) {
    rf = vector(length = length(features))
    for(i in 1:length(features)){
      f = features[i]
      .model = paste(model, f, sep="+")
      fit   = lm(as.formula(.model), d)

      # if the model can not be fit,
      # make sure the feature will not be selected
      if(sum(is.na(coef(fit)))>0){
        PRESS = NA
      } else {
        hii   = ls.diag(fit)$hat
        PRESS = sum( (fit$residual/(1-hii))^2) / nrow(d)
      }
      rf[i] = PRESS
    }
    if(all(is.na(rf))) break

    # add selected feature
    sf = features[which(rf == min(rf, na.rm=TRUE))]
    sf = sf[1]
    model = paste(model, sf, sep="+")

    # remove selected feature from list
    features = features[-which(features == sf)]

    # refit updated model to save error
    fit   = lm(as.formula(model), d)
    hii   = ls.diag(fit)$hat
    PRESS = sum( (fit$residual/(1-hii)) ^2) / nrow(d)
    PRESS_GAIN = r[r$step == step, "PRESS"] - min(rf, na.rm = TRUE)

    if(PRESS_GAIN > 0.0000001){ # > 0
      step = step + 1
      r[r$step == step, "PRESS"]   = PRESS
      r[r$step == step, "correct"] = sum((fit$residual/(1-hii))^2 <= .25) / nrow(d)
      r[r$step == step, "feature"] = sf
      r[r$step == step, "model"]   = model
    }
  }

  # plot trace(optional)
  if(plot == TRUE){
    require(lattice)
    print(xyplot(PRESS + correct ~ step, r, type="l",
      outer = TRUE, scales="free"))
  }

  model = r[r$step == step, "model"]
  fit   = lm(as.formula(model), d)

  return(list(fit = fit, trace = r))
}


########################################################################################
#####
# 8. Train/Test loop
# For each participant in data frame, determine leave-one-out crossvalidated
# prediction error for:
#  - FSR_stepAIC
#  - FSR_stepCV
#  - the Mean model (intercept only)
#  - the History model (intercept + lag 1 + lag 2)
```

```
#
# NOTE: benchmark models (Mean/History) are defined in this section

# PARAMS:
# ddd    : study data frame
# cv_list: study days to cross_validate (default: all)
train_test = function(ddd, cv_list = c(3:42)) {

  # Create result record
  # will contain the cross-validated error of the prediction of
  # each hold-out day (day 3 to 42), of each participant
  r = expand.grid(id = unique(ddd$id), days = cv_list)

  r$error_stepAIC = NA  # error of stepAIC
  r$error_stepCV  = NA  # error of LKW_cv
  r$error_mean    = NA  # error of benchmark model 1: mean
  r$error_history = NA  # error of benchmark model 2: history
  r$model_stepAIC = ""  # model found by stepAIC
  r$model_stepCV  = ""  # model found by LKW_cv (see above)

  # trace record for lkw_cv
  training_trace = data.frame()

  # for each participant
  for(p in unique(ddd$id)) {

    # get data of participant
    pdata = subset(ddd, id == p)

    # for each day in cv_list (default: all)
    for(day in cv_list){

      print(day); print(p)

      # leave one out to crossvalidate
      pdata_train = subset(pdata, stime!= day)
      pdata_test = subset(pdata, stime == day)

      # no test data? next
      if(nrow(pdata_test) != 1) next

      # not enough training data? next
      if(nrow(pdata_train) < 3) next

      #
      # FSR stepwise variable selection (stepAIC)
      result = FSR_stepAIC(pdata_train)
      model = result$fit
      predicted = predict(model, pdata_test)
      error_stepAIC = predicted - pdata_test[OUTCOME]
      r[r$id == p & r$day == day, "error_stepAIC"] = error_stepAIC
      r[r$id == p & r$day == day, "model"] = as.character(model$terms)[3]

      #
      # FSR stepwise variable selection (with PRESS)
      result = FSR_stepCV(pdata_train)
      model = result$fit
      predicted = predict(model, pdata_test)
      error_stepCV = predicted - pdata_test[OUTCOME]
      r[r$id == p & r$day == day, "error_stepCV"] = error_stepCV
      r[r$id == p & r$day == day, "model_stepAIC"] = as.character(model$terms)[3]

      # save model training trace for future analysis
      trace = subset(result$trace, !is.na(PRESS))
      if(nrow(trace) > 0){
        trace$day_cv   = rep(day, nrow(trace))
        training_trace = bind_rows(training_trace, trace)
      }
```

```
      ## Benchmark models

      # MEAN Model
      model = as.formula(paste0(OUTCOME, "~ 1"))
      predicted = predict(lm(model, pdata_train), pdata_test)
      error_mean = predicted - pdata_test[OUTCOME]
      r[r$id == p & r$day == day, "error_mean"] = error_mean

      # History Model
      model = as.formula(paste0(OUTCOME, "~ 1 + ", OUTCOME, ".l1 + ", OUTCOME,
".l2"))
      predicted     = predict(lm(model, pdata_train), pdata_test)
      error_history = predicted - pdata_test[OUTCOME]
      r[r$id == p & r$day == day, "error_history"] = error_history
    }
  }
  return(list(r = r, training_trace = training_trace))
}

results           = train_test(dd)         # feed study data into train/test loop
cv_results        = results$r              # all CV's for all models
cv_training_trace = results$training_trace # get trace for FSR_stepCV (to create plot
later)


##################################################################
# Add correctness as a prediction performance measure
# By definition, a correct prediction has a crossvalidated absolute
# residual of .5 or less

isCorrect = function(error) {
  correct = (error^2) < .25
  return(correct)
}

cv_results$correct_stepAIC = isCorrect(cv_results$error_stepAIC)
cv_results$correct_stepCV  = isCorrect(cv_results$error_stepCV)
cv_results$correct_mean    = isCorrect(cv_results$error_mean)
cv_results$correct_history = isCorrect(cv_results$error_history)


##################################################################
# 9. Save CV Results for future reference

PUBLISHED_ANALYSES[[OUTCOME]]$cv_results        = cv_results
PUBLISHED_ANALYSES[[OUTCOME]]$cv_training_trace = cv_training_trace


################################################################################
# 10. Figure 3:
# PLOT prediction for all days of a particular user (Figure 3 in the manuscript)
# two plots are shown to show to effect of crossvalidation
#  - top: without crossvalidation
#  - bottom: with crossvalidation

ID = "AS14.17" # for AS14.17, data of days 3 to 42 is available (complete record).
pdata = subset(dd, id == ID)

result = FSR_stepCV(pdata)
model = result$fit
pdata$predicted = predict(model, pdata)

pdata$observed       = unlist(pdata[OUTCOME])
cv_results           = PUBLISHED_ANALYSES[[OUTCOME]]$cv_results
crossvalidated       = subset(cv_results, id == ID)$error_stepCV
pdata$crossvalidated = pdata$observed + crossvalidated
```

```
# determine range of values on y-axis
range = range(
  c(pdata$observed, pdata$predicted, pdata$observed - 0.5, pdata$observed+0.5,
    pdata$crossvalidated)
) + c(-0.5, 0.5)


# Figure 3 Top (without cross-validation)
# NOTE: this plot does not show crossvalidated errors.
# it shows the model found, when all cases are used
# Since no crossvalidation is used, the prediction looks
# overly optimistic.

library(latticeExtra)
a = xyplot(observed + predicted ~ stime, pdata,
  col = c("black","black"),
  lty = c(2,1), pch = c(1,19), lwd = c(1.5,2), type=c("b"),
  ylab = list(OUTCOME, cex=1), xlab = list("Study Day", cex=1), cex=2,
  main = "", scales=list(cex=1), ylim= range,
  auto.key=list(text=c("Predicted", "Observed"), lines=TRUE, points=FALSE,
columns=2))

b = xyplot(I(observed+0.5) + I(observed - .5) ~ stime, pdata,
  col = c("lightgray"),
  lty = c(2), pch = c(1), lwd = c(.5), type="l",
  ylab = list("Mood", cex=1), xlab = list("Study Day", cex=1), cex=2,
  main = "", scales=list(cex=1) )
a + as.layer(b)

# Figure 3: bottom (with cross-validation)
library(latticeExtra)
a = xyplot(observed + crossvalidated ~ stime, pdata,
  col = c("black","black"),
  lty = c(2,1), pch = c(1,19), lwd = c(1.5,2), type=c("b"),
  ylab = list(OUTCOME, cex=1), xlab = list("Study Day", cex=1), cex=2,
  main = "", scales=list(cex=1), ylim= range,
  auto.key=list(text=c("Predicted (CV)", "Observed"), lines=TRUE, points=FALSE,
columns=2))

b = xyplot(I(observed+0.5) + I(observed - .5) ~ stime, pdata,
  col = c("lightgray"),
  lty = c(2), pch = c(1), lwd = c(.5), type="l",
  ylab = list("Mood", cex=1), xlab = list("Study Day", cex=1), cex=2,
  main = "", scales=list(cex=1) )
a + as.layer(b)

###################################################################
# 11. Figure 4.
# Plot training trace for a particular day to illustrate
# how performance measures develop over time, with increasing
# model complexity

cv_training_trace = PUBLISHED_ANALYSES[[OUTCOME]]$cv_training_trace

t = subset(cv_training_trace, day_cv == 8) # at day 8, traces are available for N =
27
table(t$id)
names(t)[3:4] = c("MSE", "Correct")

require(latticeExtra)
a = xyplot(MSE + Correct ~ step, groups = id, t, type=c("l"),
  lty=1, alpha=.2, lwd = 1.5, outer=TRUE, scales ="free",
  ylab = "", xlab = list(label= "Model Size (number of independent variables)",
cex=0.9))
b = xyplot(MSE + Correct ~ step, t, type=c("smooth"),
  lty=1, alpha=1, lwd = 3, outer=TRUE, scales ="free",
  ylab = "", xlab = "")
a + as.layer(b)
```

```
# To set the right font size for the figure
#update(a + as.layer(b), par.settings = list(fontsize = list(text = 20)))

###################################################################
# 12. Summarize cross-validation results
# see Table 3

# raw data
r = PUBLISHED_ANALYSES[[OUTCOME]]$cv_results
r[order(r$id, r$day), c("days", "id", "error_stepAIC", "error_stepCV", "error_mean",
"error_history")]

# summarize
summary(r)

# Prediction error plot
densityplot(~error_stepAIC, r)
densityplot(~error_stepCV, r)
densityplot(~error_mean, r)
densityplot(~error_history, r)

##
# Crossvalidated MSE
MSE = aggregate(r[c("error_stepAIC","error_stepCV","error_mean","error_history")],
list(id = r$id),
  function(x) {
    x = x[!is.na(x)]
    MSE = sum(x^2) / length(x)
    return(MSE)
  }
)

# N days per participant
N = aggregate(r[c("error_stepAIC")], list(id = r$id),
  function(x) {
    return(sum(!is.na(x)))
  }
)
names(N)[2] = "ndays"

# MEAN MSE
MSE = merge(MSE, N, by = "id")
colMeans(MSE[2:6], na.rm=TRUE) # see Table 3

# SD MSE
sds = sapply(MSE[2:6], sd, na.rm = TRUE)

# confidence interval: MEAN +/- (SEM * 1.96) (where SEM = sd/ sqrt(n) )
round(((sds / sqrt(27)) * qt(.975, 27)), 3) # half the interval.


##
# Crossvalidated Percentage Correct
# + confidence interval
correct = aggregate(
  r[c("correct_stepAIC","correct_stepCV","correct_mean","correct_history")],
  list(id = r$id),
  function(x) {
    x = x[!is.na(x)]
    perc = sum(x) / length(x)
    return(perc)
  }
)

correct = merge(correct, N, by = "id")
props = colMeans(correct[2:6], na.rm=TRUE)
props
```

```
# confidence interval
sds = sapply(correct[2:5], sd, na.rm = TRUE)
round(((sds / sqrt(27)) * qt(.975, 27)), 3) # half the interval.



#####################################################################################
# 12. Tests of significance of differences in predictive performance between
modelling
# strategies (Table 3)

# MSE
d = MSE
wilcox.test(d$error_stepAIC, d$error_mean, paired=TRUE, exact = TRUE, conf.int=TRUE)
wilcox.test(d$error_stepAIC, d$error_history, paired=TRUE, exact = TRUE,
conf.int=TRUE)

wilcox.test(d$error_stepCV, d$error_mean, paired=TRUE, exact = TRUE, conf.int=TRUE)
wilcox.test(d$error_stepCV, d$error_history, paired=TRUE, exact = TRUE,
conf.int=TRUE)

# % Correct
c = correct
wilcox.test(c$correct_stepAIC, c$correct_mean, paired=TRUE, exact=TRUE,
conf.int=TRUE)
wilcox.test(c$correct_stepAIC, c$correct_history, paired=TRUE, exact=TRUE,
conf.int=TRUE)

wilcox.test(c$correct_stepCV, c$correct_mean, paired=TRUE, exact=TRUE, conf.int=TRUE)
wilcox.test(c$correct_stepCV, c$correct_history, paired=TRUE, exact=TRUE,
conf.int=TRUE)


#####################################################################################
# 13. Incremental test
#

# trace record
inc_results = data.frame()
for(max_days in 8:42){
  print(max_days)
  inc_dd = subset(dd, stime <= max_days)
  results = train_test(inc_dd, cv_list = max_days)
  r = results$r
  r$day_cv = max_days
  inc_results = rbind(inc_results, r)
}

#####################################################################################
# 14. Save results of incremental test for future reference

PUBLISHED_ANALYSES[[OUTCOME]]$inc_results = inc_results


#####################################################################################
# 16. Preprocess and summarise results

inc_results = PUBLISHED_ANALYSES[[OUTCOME]]$inc_results

# remove days with missing result data
inc_results = subset(inc_results, !is.na(error_stepAIC))

# calc % correct
inc_results$correct_stepAIC = isCorrect(inc_results$error_stepAIC)
inc_results$correct_stepCV  = isCorrect(inc_results$error_stepCV)
inc_results$correct_mean    = isCorrect(inc_results$error_mean)
```

```
inc_results$correct_history = isCorrect(inc_results$error_history)

# number of predicted days per participant
table(inc_results$id)

# Remove one extreme MSE outlier (mood)
if(any(inc_results$error_stepAIC > 100))
  inc_results = inc_results[-which(inc_results$error_stepAIC > 100), ]

# MSE
MSE = aggregate(inc_results[c("error_stepAIC", "error_stepCV", "error_mean",
"error_history")],
  by = list(day = inc_results$day_cv),
  function(x) {
    x = x[!is.na(x)]
    MSE = sum(x^2) / length(x)
    return(MSE)
  })

# % Correct
correct = aggregate(inc_results[c("correct_stepAIC", "correct_stepCV",
"correct_mean", "correct_history")],
  by = list(day = inc_results$day_cv),
  function(x) {
    x = x[!is.na(x)]
    x = sum(x) / length(x)
    return(x)
  })


#####################################################################################
# 17. Does relative predictive performance of stepAIC and stepCV increase over time
# compared to intercept only model performance? (table 4)

testINC = function(MSE, correct, outcome, comparison){
  # MSE
  MSE$diff = MSE[[paste0("error_", outcome)]] - MSE[[paste0("error_", comparison)]]
  fitMSE = lm(diff ~ I(day-8), MSE)
  print(summary(fitMSE))

  p = xyplot(diff ~ day, MSE, type=c("p", "r"), lwd = (2), # ylim = c(-0.4,0.4),
    ylab = "MSE \n (difference with mean model)", cex=1.2,
    xlab = "Study Day",
    panel = function(x,y, ...) {
      panel.abline(h = 0, lty = 2, alpha=.5)
      panel.xyplot(x,y, ...)
    })
  print(p)

  # Correct
  correct$diff = correct[[paste0("correct_", outcome)]] - correct[[paste0("correct_",
comparison)]]
  correct$diff = correct$diff * 100 # %
  fitCorrect = lm(diff ~ I(day-8), correct)
  print(summary(fitCorrect))

  p = xyplot(diff ~ day, correct, type=c("p", "r"), lwd = (2), # ylim = c(-0.4,0.4),
    ylab = "% Correct\n (difference with mean model)", cex=1.2,
    xlab = "Study Day",
    panel = function(x,y, ...) {
      panel.abline(h = 0, lty = 2, alpha=.5)
      panel.xyplot(x,y, ...)
    })
  print(p)

  return(list(MSE = fitMSE, correct = fitCorrect))
}
```

```
stepAIC_inctest = testINC(MSE, correct, "stepAIC", "mean")
stepCV_inctest  = testINC(MSE, correct, "stepCV", "mean")


##################################################################################
# 18. Figure 5
# Plot of comparative performance of stepAIC and stepC, compared to Mean model.

MSE$stepAIC = MSE$error_stepAIC - MSE$error_mean
MSE$stepCV  = MSE$error_stepCV - MSE$error_mean

xyplot(stepAIC + stepCV ~ day, MSE, type=c("p", "r"), lwd = (2), # ylim = c(-0.5,2),
  ylab = "MSE \n (difference with mean model)",
  xlab = "Study Day", cex = 1.2,
  outer=TRUE, layout = c(1,2), panel = function(x,y, ...) {
    panel.abline(h = 0, lty = 2, alpha=.5)
    panel.xyplot(x,y, ...)
  })

correct$stepAIC = (correct$correct_stepAIC - correct$correct_mean) * 100
correct$stepCV  = (correct$correct_stepCV - correct$correct_mean) * 100

xyplot(stepAIC + stepCV ~ day, correct, type=c("p", "r"), lwd = (2), # ylim = c(-
0.4,0.4),
  ylab = "% Correct  \n (difference with mean model)", cex=1.2,
  xlab = "Study Day",
  outer=TRUE, layout = c(1,2), panel = function(x,y, ...) {
    panel.abline(h = 0, lty = 2, alpha=.5)
    panel.xyplot(x,y, ...)
  })


#####################################################################################
# 19. write analysis results to disk
save(file = paste0(wd, "PUBLISHED_ANALYSES.RDA"), PUBLISHED_ANALYSES)
```
